# The Value of Trivial Symptoms

**Published:** 1890-07

**Authors:** G. M. Pease

**Affiliations:** San Francisco, Cal.


### "THE VALUE OF TRIVIAL SYMPTOMS."

G. M. PEASE, M. D., SAN FRANCISCO, CAL.

Under this heading, on page 146, April number of THE HOMŒOPATHIC PHYSICIAN, are some fine thoughts.

The first paragraph I would like to have considered as a prelude to the report of a case which was under treatment some years ago.

A gentleman had been a sufferer for fifteen years from intermittent fever. He had tried several physicians of both schools, without the slightest benefit, and although he had been pronounced incurable he had hopes to the contrary. Under such circumstances unusual care was taken in writing out his case, that no point should be lost in its study. After six months no improvement was manifest, and, losing courage, I felt obliged to acknowledge defeat, but he insisted upon further trial.

The patient had been closely questioned many times, with the hope that some new and, perhaps, guiding symptom might be found.

Now it was proposed to regard him as if seen for the first time.

I suggested that in his recital he begin at the head and go down to his toes. At the mention of toes he was reminded of what, he thought, was of no account, though the symptom had existed for years. When he took off his boots *at night* the ball of the right great toe invariably itched.

In those days we did not have the many repertories that now assist us, but I remembered to have seen somewhere in my readings this apparently absurd symptom, and without further delay a search was begun which resulted in finding the almost identical symptom under Natrum-sulphuricum. Nearly every other symptom was pictured under this remedy, and a rapid *cure* followed its administration, and two persons, patient and doctor, felt like putting a big feather in the cap of Homœopathy.

